# School outbreak of coxsackievirus A16 in Antipolo City, Philippines, October 2022

**DOI:** 10.5365/wpsar.2025.16.4.1212

**Published:** 2025-12-16

**Authors:** Daniel SP Garcia, Alireza S Faiyaz MF,, Nino D Rebato, Mariz Zheila Blanco-Payuyo, John Bobbie Roca, Concepcion G Lat

**Affiliations:** a Field Epidemiology Training Program, Intermediate Course, City Epidemiology, Health Statistics, Disaster and Response Unit, Antipolo City Health Office, Rizal, Philippines.; b City Epidemiology, Health Statistics, Disaster and Response Unit, Antipolo City Health Office, Rizal, Philippines.; c Municipal Human Resources Management, Government of San Jose de Buan, Samar, Philippines.; d Regional Epidemiology Surveillance Unit, Center for Health and Development Region 4A, Department of Health, Manila, Philippines.; e Antipolo City Health Office, Rizal, Philippines.

## Abstract

**Objective:**

An investigation team was deployed to determine the cause of an outbreak of a cluster of cases of fever and rash in a public elementary school in Antipolo City, Philippines, after the Public Health Unit was notified on 24 October 2022. The team also aimed to identify the source of the outbreak and to guide prevention measures.

**Methods:**

Active case-finding for hand, foot and mouth disease was conducted at the school. A suspected case was defined as any learner who developed acute febrile illness with a papulovesicular rash on the palms and soles of the feet during 16–30 October 2022. Interviews with key informants were conducted and included school staff and parents. Oropharyngeal swabs were collected for polymerase chain reaction (PCR) testing.

**Results:**

Nineteen suspected cases of hand, foot and mouth disease were detected, predominantly in grade 1 learners (16, 84%). Most cases (14, 74%) were 6 years old, and just over half were male (11, 58%). The first case occurred in a 6-year-old in grade 1 who attended class with a papulovesicular rash. Twelve learners (63%) from the same section developed symptoms, two of whom were seatmates of the first case. Two out of the 10 swabs collected were tested by PCR, both of which were positive for coxsackievirus A16.

**Discussion:**

The causative agent of this outbreak was identified as coxsackievirus A16. Disease transmission occurred through close contact with the index case and possibly through shared classroom objects. Follow-up actions included dissemination of a memorandum about preventing the disease to all public elementary and secondary schools that emphasized symptom screening (i.e. for fever and rash), self-isolation at the onset of symptoms, regular disinfection of classroom surfaces and regular handwashing, especially before and after eating.

Hand, foot and mouth disease (HFMD) is an acute, infectious disease that mostly affects children worldwide. Caused by coxsackievirus A16 (CV-A16) and other enteroviruses, HFMD spreads quickly, causing outbreaks that can lead to closures of nurseries, day cares and schools. (*1*) Symptoms include low-grade fever, mouth sores and rashes, which are commonly found on the hands and feet. This disease is highly contagious, and cases can remain infectious for weeks after symptoms have resolved. (*2*) Asymptomatic individuals can also transmit the virus. (*1*)

In south-eastern Asia, where the disease is endemic, outbreaks are commonly caused by CV-A16. Case numbers tend to surge during the middle of the year, especially when the humidity is high. (*3*) In the Philippines, HFMD is an immediately notifiable disease and must be reported promptly to public health authorities. In 2022, the Philippines Department of Health reported a total of 3365 cases of HFMD distributed across the country. (*4*)

On 24 October 2022, the Public Health Unit of Antipolo City was notified of a cluster of cases of fever and rash in a public elementary school. The following day, a team of field epidemiologists was deployed to investigate the health event to determine the cause of the cluster and the likely transmission route. A secondary objective was to provide recommendations for controlling the outbreak and for preventing future outbreaks.

## Methods

### Study setting

Antipolo City is located in Rizal province, Philippines, and has a population of 887 399. (*5*) A total of 10 200 learners attended the elementary school involved in the outbreak. The school has adopted a double-shift schedule for classes (i.e. children attend either in the mornings or afternoons) and accommodates around 5000 learners per shift, with an average class size of 50. (*6*) At the time of the outbreak, there were 1591 learners in grade 1 distributed between 18 classrooms. Each classroom had two sections, and pupils spent most of their time in the same classroom and section.

### Study design and case definitions

On 25 October 2022, members of the outbreak investigation team visited the school and conducted active case-finding activities. A suspected case was defined as any learner who developed or had developed acute febrile illness with a papulovesicular rash on the palms and soles of the feet during the period 16–30 October 2022. A confirmed case was a suspected case with positive laboratory results for human enteroviruses that cause HFMD. (*7*) An epidemiologically linked case was a suspected case whose illness was not laboratory-confirmed, but whose symptoms were temporally and geographically related to a laboratory-confirmed case or who was in a chain of transmission with another epidemiologically linked case.

### Data collection and analysis

Parents and guardians of suspected cases provided consent before being interviewed using a structured questionnaire to collect demographic data and information about clinical symptoms and exposure history. The school nurse and class adviser were also interviewed to gather information about learners’ seating arrangements, school sanitation and hygiene protocols, and children’s behaviour during their time at school. The head of the City Epidemiology, Health Statistics, Disaster and Response Unit (CESDRU) of the Antipolo City Health Office was interviewed to determine whether other cases of HFMD had occurred in the city. The medical records of the cases who consulted the village health centre in the school’s catchment area during 16–30 October 2022 were reviewed. School clinic records were also reviewed. The investigation team assessed learners' proximity to and use of points of entry and exit. Sanitary facilities were also inspected. Data were managed using Microsoft 365 Excel 2021.

For a descriptive analysis, frequencies and percentages were used to express the demographic characteristics of HFMD cases, and their grade, classroom section, date of onset, signs and symptoms, and history of travel. An epidemic curve, based on the date of symptom onset, was generated to illustrate the epidemiological connections between cases. A spot map, based on information about learners’ movements, was created to understand transmission patterns and factors that may have contributed to the spread of disease.

### Laboratory investigations

Oropharyngeal swabs were collected from suspected cases and preserved using BioSci Universal Transport Medium (Shenzen Dakewe Bio-engineering Co., Shenzen, China) before testing at the National Reference Laboratory. The specimens were tested for enteroviruses and coxsackievirus using semi-nested polymerase chain reaction (PCR), with a detection target of the 5′ untranslated region.

## Results

### Descriptive analysis

**Fig. 1:**
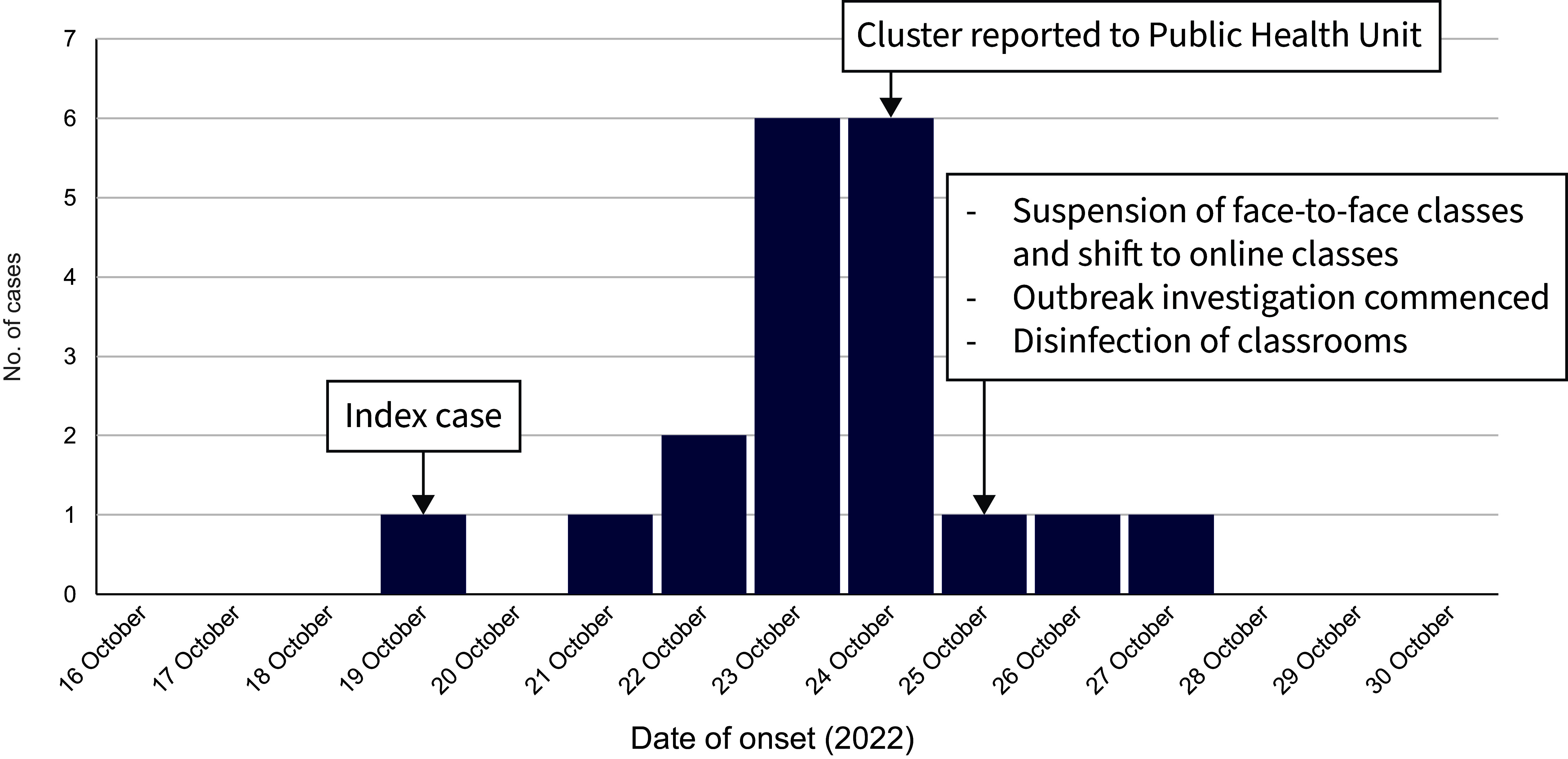
Epidemiological curve of cases of hand, foot and mouth disease at a public elementary school, by date of onset, Antipolo City, Rizal province, Philippines, 16–30 October 2022 (*N* = 19)

A total of 19 suspected cases of HFMD were identified. The earliest date of symptom onset was 19 October and the latest was 27 October; the peak occurred on 23–24 October (**Fig. 1**). Case numbers decreased after 25 October, when face-to-face classes were suspended and teaching shifted to online and modular learning. All cases presented with fever and papulovesicular rash on the palms and soles of the feet.

[**insert** Figure 1]

**Fig. 2:**
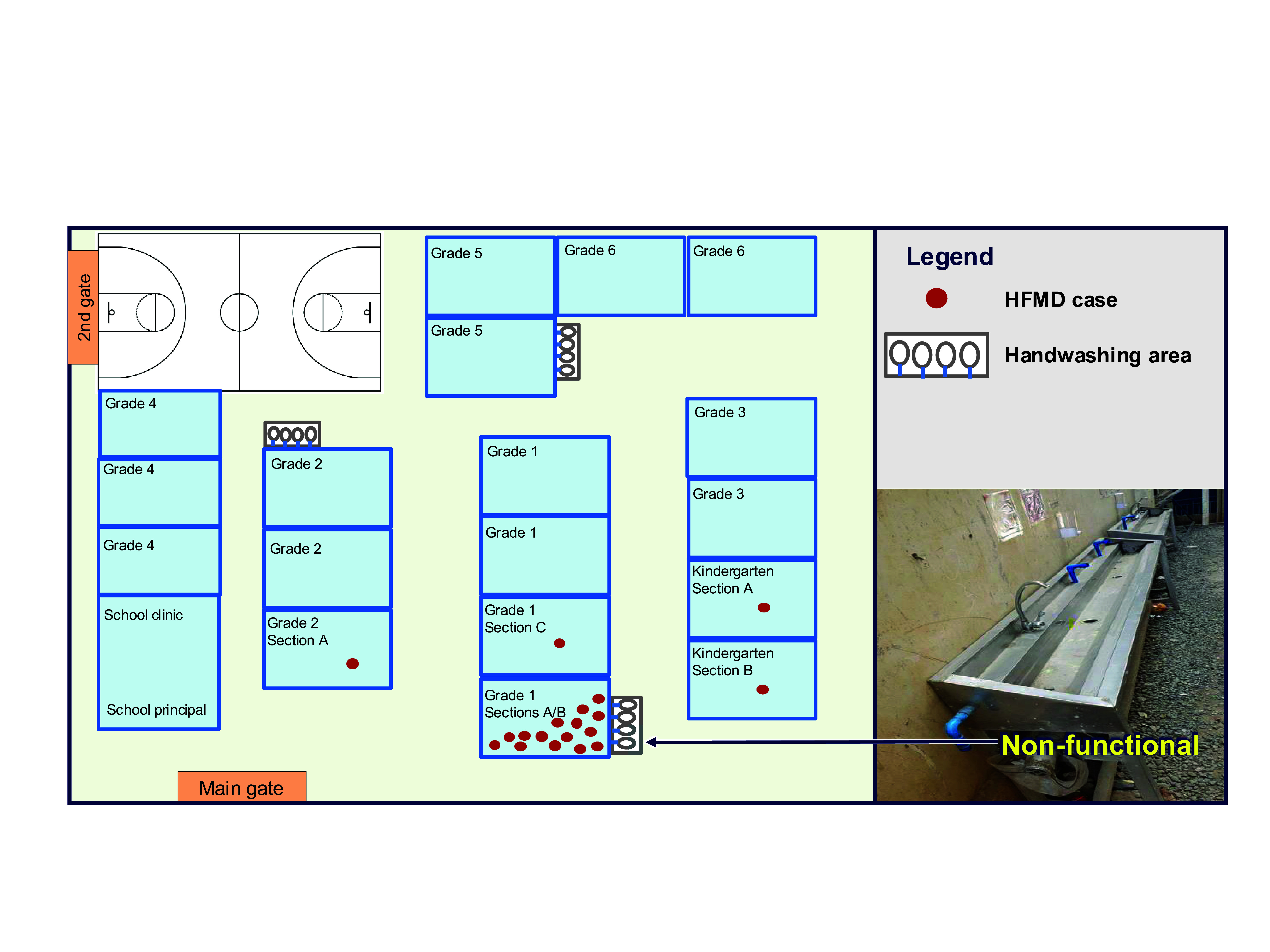
Spot map of cases of hand, foot and mouth disease in a public elementary school, Antipolo City, Rizal province, Philippines, 16–30 October 2022 (*N* = 19)^a^

The index case was a 6-year-old female first grader in section A who attended school in the mornings and whose symptoms were first noticed on 19 October 2022. She had attended mass at a nearby church 3 days before symptom onset. No other travel history was noted, and there were no other cases of HFMD at her home or in her community. Twelve of the suspected cases were from the same school section as the index case, and two of them had sat next to the index case. Two first graders from section B’s afternoon shift who had used the same armchair as the index case also subsequently developed symptoms (**Fig. 2**).

[**insert** Figure 2]

Grade 1 learners (i.e. those aged 5 to 6 years) accounted for the greatest proportion of cases (16, 84%). Just over half were male (11, 58%), and the majority were aged 6 years (14, 74%) (**Table 1**). In addition to fever and papulovesicular rash, the majority experienced asthenia, or general weakness (16, 84%), and around half (10, 53%) had mouth sores. A smaller number had headaches or abdominal pain (8 each, 42%), and 4 (21%) experienced other signs and symptoms, including loss of appetite, nausea or vomiting (**Table 1**). Grade 1 section A had the highest attack rate (13/44, 30%), followed by grade 1 section B (2/46, 4%) (**Table 1**). A review of school records revealed no reports of HFMD during the past 5 years.

**Table 1:** Characteristics of cases of hand, foot and mouth disease at a public elementary school, Antipolo City, Rizal province, Philippines, 16–30 October 2022

Characteristic	No. (%) of cases (*n* = 19)	No. (%) of males (*n* = 11)	No. (%) of females (*n* = 8)
**Age (years)**			
**5**	**2 (11)**	**2 (18)**	**0 (0)**
**6**	**14 (74)**	**7 (64)**	**7 (88)**
**7**	**2 (11)**	**1 (9)**	**1 (13)**
**8**	**1 (5)**	**1 (9)**	**0 (0)**
**Signs and symptoms^a^**			
**Fever**	**19 (100)**	**11 (100)**	**8 (100)**
**Papulovesicular rash**	**19 (100)**	**11 (100)**	**8 (100)**
**Asthenia (general weakness)**	**16 (84)**	**11 (100)**	**5 (63)**
**Mouth sores**	**10 (53)**	**8 (73)**	**2 (25)**
**Headache**	**8 (42)**	**6 (55)**	**2 (25)**
**Abdominal pain**	**8 (42)**	**5 (45)**	**3 (38)**
**Other^b^**	**4 (21)**	**2 (18)**	**2 (25)**
**Grade and section (no. of learners)^c^**			
**Kindergarten**			
**Section A (***n* **= 34)**	**1 (3)**	**1 (3)**	**0 (0)**
**Section B (***n* **= 31)**	**1 (3)**	**1 (3)**	**0 (0)**
**Grade 1**			
**Section A (***n* **= 44)**	**13 (30)**	**7 (16)**	**6 (14)**
**Section B (***n* **= 46)**	**2 (4)**	**1 (2)**	**1 (2)**
**Section C (***n* **= 48)**	**1 (2)**	**0 (0)**	**1 (2)**
**Grade 2**			
**Section A (***n* **= 45)**	**1 (2)**	**1 (2)**	**0 (0)**

^a^ Interviewees could provide multiple responses.

^b^ This category includes loss of appetite, nausea and vomiting.

^c^ These values are the attack rate, calculated as a percentage (the number of cases/the total number of learners).

### Key informant interviews

The mother of the index case was unaware of the signs and symptoms of HFMD and let her child attend school thinking it was an ordinary rash. According to the class adviser and the school nurse, all learners had their temperature checked as they entered the classroom (before classes started at 06:00). However, children were not routinely screened for other symptoms. According to CESDRU, no outbreaks of HFMD had been previously identified or detected in the city.

### Environmental inspection and laboratory testing

Environmental inspection revealed inadequate handwashing areas, with leaks rendering some facilities nonfunctional. The assigned handwashing area for grade 1 section A was not functional due to a leaking pipe (**Fig. 2**). Learners were observed failing to wash their hands regularly. Few learners used alcohol-based sanitizer inside the classroom for hand hygiene before and after eating. During break time, learners were seen pushing and hugging one another, and running inside and outside the classrooms. Most of the learners engaging in these behaviours were males.

A total of 10 specimens were collected and two were tested by PCR. Both of these samples, which were from seatmates of the index case, were positive for CV-A16. The remaining eight samples were not tested, and these cases were classified as epidemiologically linked.

## Discussion

Our investigation confirmed that the cluster of cases of fever and rash in a public elementary school in Antipolo City, Rizal province, Philippines, were HFMD caused by CV-A16. The outbreak was traced to a learner in grade 1, and disease transmission occurred through close contact with the index case and possibly through shared classroom objects. A review of records and interviews with CESDRU staff suggested that this event constituted the city’s first report of HFMD in a public school setting.

Our investigation indicated that insufficient hand hygiene practices and a lack of symptom screening allowed the virus to spread unchecked within the school. Because learners were not regularly screened for rash at the school gate and when they entered their classrooms, the index case was able to attend classes while she had a papulovesicular rash but was afebrile. According to an HFMD rapid evidence review, people with the disease are most contagious during the first week of infection. (*8*)

All cases had mild and self-limiting symptoms, consistent with coxsackievirus infections. Studies conducted in China, southern Viet Nam and Pangasinan province in the Philippines have previously also identified CV-A16 as the cause of HFMD outbreaks in which the majority of affected individuals had only mild symptoms. (*9*-*11*) In contrast, more severe presentations of HFMD are more commonly linked to infection with other enteroviruses.

The index case was from grade 1 section A, which had the highest attack rate. Our spot map analysis revealed that the two cases who tested positive for CV-A16 were seatmates of the index case. Moreover, the two cases from the school’s afternoon shift used the same chair as earlier-onset cases from the morning shift. Collectively, these patterns suggest transmission via an inanimate object. Previous studies, including one in a childcare centre in China and another in a kindergarten in Hong Kong Special Administrative Region (China), have established that HFMD can be transmitted through contact with contaminated objects. (*12*, *13*) Coxsackieviruses can remain viable and infective on hard, nonporous surfaces, including chairs and tables, for up to 2 weeks. (*14*) Inadequate disinfection of surfaces that are frequently touched, poor handwashing practices, and the close-contact nature of children’s behaviours likely played roles in the transmission of HFMD in this setting. The unknown source of exposure for the index case implies asymptomatic transmission, aligning with findings from a study in China in which HFMD was spread by individuals without symptoms. (*15*)

This study had several limitations. Being descriptive, it does not test a hypothesis or identify risk factors. Another limitation is the low testing rate; only two out of 10 specimens were tested due to resource constraints. Nevertheless, the two positive results coupled with the consistent clinical presentation and strong epidemiological linkage among cases were considered sufficient evidence to identify the responsible pathogen as CV-A16.

Actions taken to control the spread of the outbreak in the school – a shift to online and modular learning in the affected classes, prompt screening for symptoms, isolation at the onset of symptoms, disinfection of surfaces and ensuring frequent handwashing – proved effective, and no new cases were reported in the school after 27 October. However, 1 month later, a cluster of new cases occurred at another elementary school in the same province.

In the wake of this investigation, the team made a series of recommendations – aimed at both schools and local authorities – for improving control of and preventing HFMD. For schools, the recommendations included screening learners for symptoms (i.e. fever and rash) when they enter their classroom, disinfecting frequently touched surfaces and objects, and implementing strict rules about handwashing. The team also recommended that the local government of Antipolo City promote health education, strengthen community detection of HFMD and advocate for self-isolation at symptom onset.

In further follow-up action, a public health report was submitted by CESDRU to the Department of Education in Antipolo City. This led to the creation and dissemination of a departmental memorandum to reinforce awareness and prevention of HFMD and the provision of functional handwashing facilities in all public elementary and secondary schools. The memorandum also directed the Department of Education to facilitate a symposium about preventing HFMD for parents at all schools. In a more recent initiative, started in 2024, the Public Health Unit and school authorities are working jointly to establish a digitized symptom-based disease surveillance system in schools.

In conclusion, our investigation and subsequent actions underscore the importance of rapid containment, health education and sustained public health collaboration to effectively respond to HFMD outbreaks in school settings. Ensuring coordinated efforts between schools and local health authorities is crucial to maintaining proactive and prevention-oriented school and health systems.
